# Impact of screw reinsertion on osteosynthesis stability in Schatzker IV tibial plateau fractures: a biomechanical study

**DOI:** 10.1051/sicotj/2025008

**Published:** 2025-02-27

**Authors:** Shuhei Hiyama, Tsuneari Takahashi, Jiro Ando, Yoshiya Nibe, Tomohiro Matsumura, Katsushi Takeshita

**Affiliations:** 1 Department of Orthopaedics, School of Medicine, Jichi Medical University 3311-1 Yakushiji Shimotsuke 329-0498 Japan; 2 Department of Emergency and Critical Care Medicine, Jichi Medical University Shimotsuke Japan

**Keywords:** Biomechanical study, Medial tibial plateau fracture, Screw reinsertion, Locking plate, Porcine tibia

## Abstract

*Introduction*: This biomechanical study evaluated the effect of screw reinsertion with a locking plate on fixation strength and the stability of osteosynthesis in medial tibial plateau fractures using porcine bone. *Materials and methods*: Thirty porcine tibiae were divided into three groups: group A (underwent biomechanical testing after medial tibial fixation with a large fragment T-shaped locking plate), group B (underwent plate fixation, followed by the removal of all screws and plates and refixation with the same screws and plates using the same holes before biomechanical testing), and group C (underwent biomechanical testing once after plate fixation, followed by the removal of all screws and plates, refixation with the same screws and plates using the same holes, and then biomechanical testing). The translation pattern of the constructs in each group was examined using cyclic loading tests. The changes in the joint gap and step-off after 2000 cycles were compared among the three groups. *Results*: Significant differences in displacement were observed at 10–100 cycles (group A: −0.01 ± 0.04 mm, group B: −0.02 ± 0.04 mm, group C: −0.13 ± 0.15 mm, *P* = 0.021). However, no significant differences were found in other displacement and translation measurements among the groups. Regarding the gap and step-off among groups, significant differences were observed in anterior and posterior gap changes. Despite the statistical significance, the absolute displacement values were small, suggesting minimal clinical relevance. These findings indicate that reinserting screws and plates into the same hole may not substantially compromise overall fixation strength. *Conclusion*: Screw reinsertion in the same holes after removal did not significantly compromise the stability of osteosynthesis in this biomechanical model. These findings suggest that reinsertion may be a viable option in revision surgery.

## Introduction

The incidence of posttraumatic knee osteoarthritis (KOA) after tibial plateau fractures is high [1]. To prevent KOA, plates, and screws are used to stabilize tibial plateau fractures. However, some fractures are associated with severe damage to the overlying soft tissue. Bicondylar fractures, fracture dislocations, and shaft avulsions typically have the worst soft tissue injuries. These high-energy fracture patterns may require surgery with large implants; however, because of the soft tissue damage, surgery is associated with a potential risk of wound complications [2]. Cortical (nonlocking) screws play an essential role in osteosynthesis. The insertion torque of these screws is related to the success of interfragmentary fixation and nonlocking plate (LP) and screw constructs [3]. In osteosynthesis, screws frequently require revision if surgical site infection (SSI) occurs. Moreover, regardless of whether the bone union has been achieved, the surgeon will consider implant removal. If the infection has healed, the surgeon may consider operating with the same size LP and the same hole. Nevertheless, concerns exist about reduced purchase and diminished pullout strength when screws are reinserted into the same drill hole [4]. A previous study reported that perioperative reinsertion of the same screw or a larger diameter screw through the same trajectory, after adjusting the screw trajectory, did not affect the pullout strength of the screw [5]. However, this study focused on pedicle screws, making it difficult to directly apply its findings to the mechanical strength of screw reinsertion in LP. To the best of our knowledge, no studies have investigated the effect of screw reinsertion with an LP on osteosynthesis stability. We hypothesized that screw reinsertion into the same holes would decrease fixation stability, leading to increased displacement under cyclic loading. Therefore, we compared the biomechanical properties of plate osteosynthesis in porcine bone with and without screw reinsertion in medial placement. This study aimed to quantify the potential impact of screw reinsertion with LP on bone joint stability.

Since human cadaveric studies pose ethical and logistical challenges, porcine bone was selected as an alternative model for biomechanical similarity to human bone. In adult specimens, porcine bone exhibits a high similarity to human bone in terms of density and trabecular bone structure. Compared to human bone, porcine bone often demonstrates similar values for elastic modules and compressive strength, making it suitable for orthopaedic research. Additionally, long bones such as the tibia and femur of porcine possess bone density comparable to that of human bodies [6, 7]. Furthermore, compared to human cadaveric or excised bones, porcine bones are more affordable and can be consistently sourced.

## Materials and methods

### Study design

**Figure 1:**
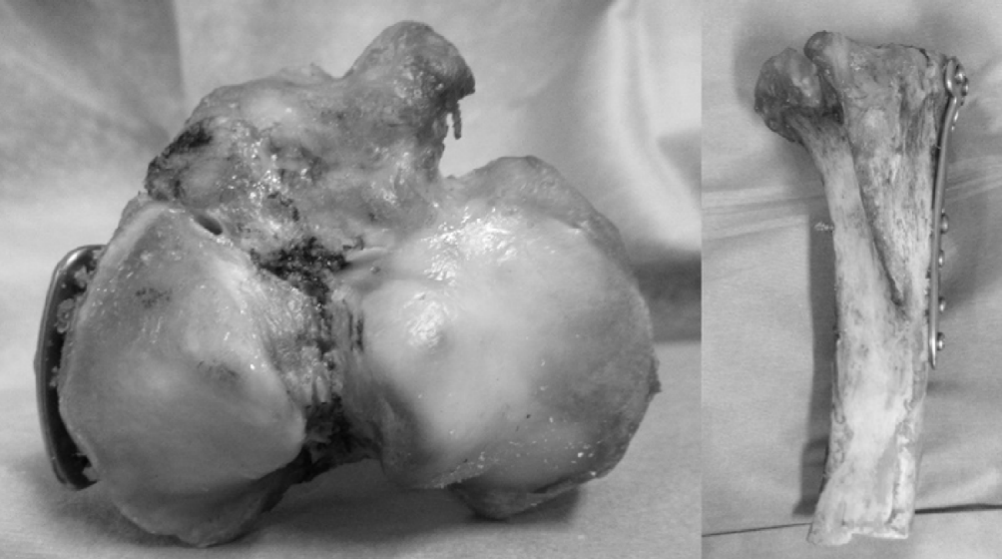
Illustration of the fracture line and plate position in a right knee specimen. The placement of the fixation plate relative to the fracture site is shown.

The animal experiments were conducted in the institution’s biomechanics laboratory following the regulations of the Institutional Animal Care and Use Committee. As this was an ex vivo study, ethical approval was waived by the same committee. Thirty fresh porcine tibiae from animals (6 months old, weighing 100–120 kg, Tokyo Shibaura Zouki, Japan) were used. All specimens were selected from tibia of similar size to minimize variability. The specimens were thawed at room temperature for at least 24 h prior to use. Each specimen was modeled with medial tibial plateau fractures (MTPFs) and fixed using a large fragment T-shaped LP (TOMOFIX, DePuy Synthes, Zuchwil, Switzerland) with a single cortex screw and six locking screws. The plate attachment was fixed to the medial tibia so that the direction of the central screw hole in the proximal tibia was 0° (medial position) from the transverse diameter of the tibial plateau (Figure 1).

It is known that there is a difference in fixation strength between medial and anteromedial [8]. In this study, medial was chosen because it provides higher fixation strength, allowing the evaluation to focus solely on the impact of reinsertion.

**Figure 2:**
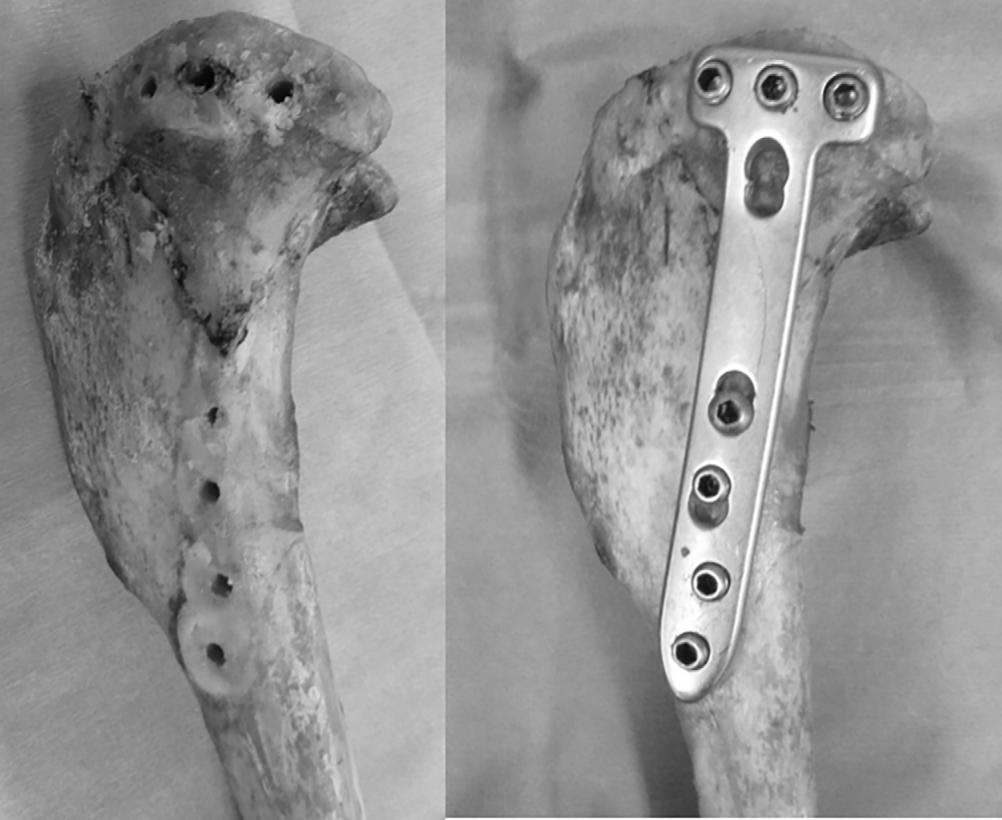
Specimen after removal of all screws and the plate, followed by re-fixation using the same screw holes. This simulates the condition of reinsertion in clinical settings.

The specimens were divided into three groups, with each group comprising 10 specimens. Group A underwent biomechanical testing after medial tibial fixation using a large fragment T-shaped LP. Group B underwent plate fixation, followed by the removal of all screws and plates and refixation with the same plates and screws using the same holes before biomechanical testing. Group C underwent biomechanical testing once after plate fixation, followed by the removal of all screws and plates, refixation with the same screws and plates using the same holes, and then biomechanical testing (Figure 2).

### Biomechanical assessment

**Figure 3:**
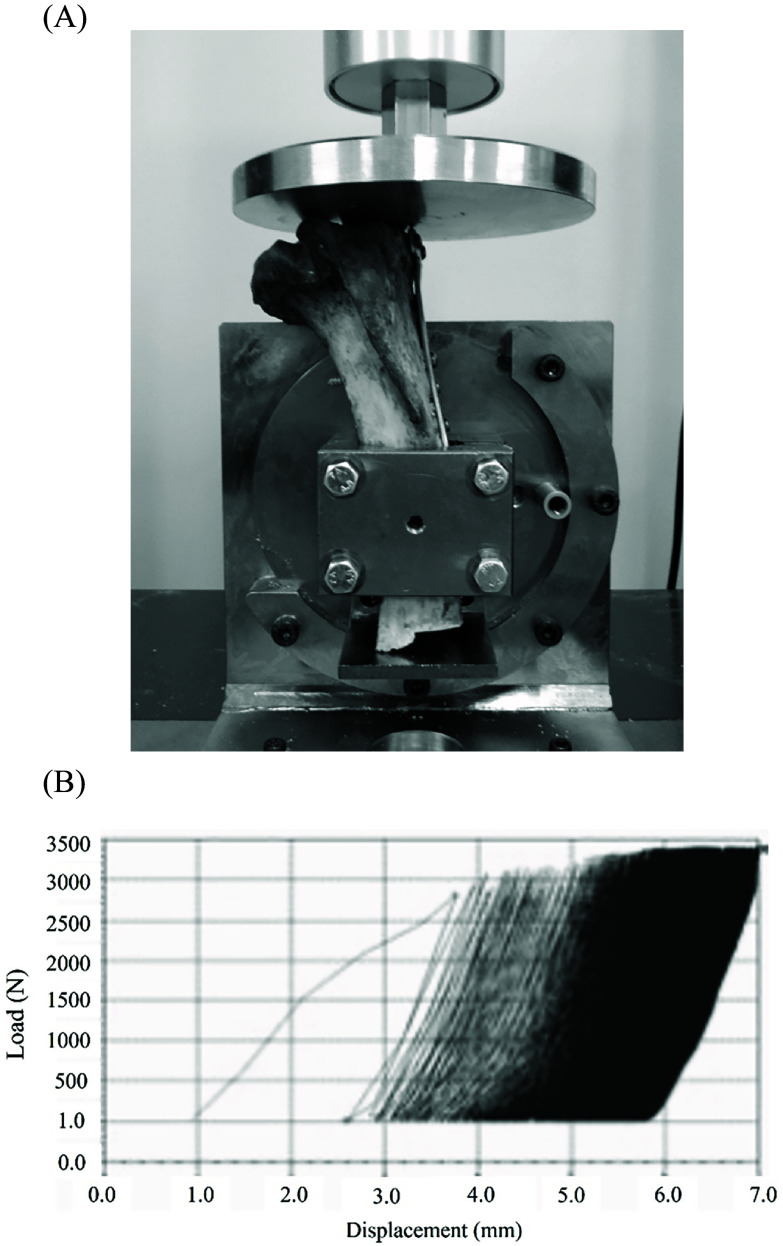
Tensile testing setup and load–displacement curve during cyclic testing. (A) Photograph of the tibia mounted in the tensile testing machine after internal fixation with a locking plate. (B) Schematic representation of the load–displacement curve generated by the software during cyclic loading. An axial load of 2800 N was applied to the tibial joint surface over 2000 cycles to evaluate biomechanical stability.

The translation pattern of the fixed constructs was examined using cyclic loading tests. A tensile testing machine (Tensilon RTG-1310; Orientec Corporation, Tokyo, Japan) equipped with a specially designed grip set was used to apply cyclic loads to the prepared constructs along the postoperative mechanical axis of the tibia [8–10]. Moreover, the 4 cm distal end of the tibia was clamped with a custom-made jig and set so that the articular surface was perpendicular to the loading direction (Figure 3A). Excess cartilage and bone from the intercondylar margin of the tibia were excised to apply load to the medial and lateral articular surfaces [8]. Then, a repetitive load of up to 2800 N was applied to the tibia (2000 cycles, 0.5 Hz). The loading conditions were determined in accordance with the methods of previous studies [8, 11]. The force transmitted to the knee joint during normal walking is two to three times the body weight. Therefore, the test protocol was used to simulate the gait of a healthy person weighing 70 kg for 10–100, 100–500, 500–1000, 1000–1500, and 1500–2000 cycles. The displacement in the direction of the mechanical axis during the cycles was determined at 100. The software was used to calculate the displacement of the actuator at 1, 500, 1000, 1500, and 2000 cycles (Figure 3B). The changes in the gaps and steps at the anterior, central, and posterior fracture sites after cyclic loading tests were measured using precision calipers with an accuracy of 0.1 mm. The anterior, central, and posterior fracture sites were defined as the anterior edge, central half, and posterior edge of the articular surface, respectively. The step-off was defined as the value of the sinking of the medial fracture fragment relative to the articular surface [8]. We defined fixation failure as either plate or screw breakage or displacement/translation exceeding 5 mm [12].

### Statistical analysis

Differences between groups were evaluated using one-way analysis of variance (ANOVA) with Tukey’s post hoc analysis. The gaps and step-offs of the fracture site were measured thrice and compared using their median values. All data are presented as the mean ± standard deviations. Statistical significance was considered at *P* < 0.05.

A priori power analysis was performed using G*Power 3.1 (Franz Paul, Kiel, Germany) [13]. The sample size for α error was set at <0.05, for β error at <0.20, and the effect size was 0.46 for repeated-measures ANOVA and 0.60 for one-way ANOVA. All statistical data were analyzed using EZR software [14]. The minimum sample size by one-way ANOVA of changes in gaps and step-offs of the fracture site was 66. If a significant difference was found with fewer samples than these, it meant that the difference was quite large. Therefore, the effect sizes for the repeated measures and one-way ANOVA were larger than Cohen’s effect size guidelines, indicating that the differences between each group were highly significant.

## Results

### Displacement and translation during cyclic loading

The mean displacement and translation results are shown in Tables 1 and 2, respectively. One-way ANOVA revealed a significant difference in displacement during cyclic loading in the 10–100 intervals (group A: −0.01 ± 0.04, group B: −0.02 ± 0.04, group C: −0.13 ± 0.15, *P* = 0.021). Post hoc analysis also revealed that the mean displacement during the 10–100 intervals of group A was lower than that of groups B and C (*P* = 0.048 and 0.032, respectively). No significant differences were found in all other displacement and translation measurements among the three groups in any category (Tables 1 and 2).

**Table 1:** Mean displacement (mm) during cyclic loading for each group. Displacement represents the relative movement of the fixation construct under repetitive loading, which may indicate its stability. A smaller displacement suggests better fixation strength and resistance to loosening.

Intervals	Mean displacement in mm			
	Group A (*n* = 10)	Group B (*n* = 10)	Group C (*n* = 10)	*P*-value
10–100 cycles	−0.01 ± 0.04	−0.02 ± 0.04	−0.13 ± 0.15	0.021
100–500 cycles	0.00 ± 0.06	−0.02 ± 0.08	−0.02 ± 0.04	0.613
500–1000 cycles	0.02 ± 0.03	0.01 ± 0.04	−0.00 ± 0.02	0.357
1000–1500 cycles	−0.01 ± 0.05	0.02 ± 0.04	0.00 ± 0.01	0.318
1500–2000 cycles	−0.03 ± 0.04	−0.01 ± 0.02	−0.02 ± 0.01	0.154

**Table 2:** Mean translation (mm) during cyclic loading for each group. Translation reflects the shift of the fixation construct under cyclic loading, which can impact postoperative stability. The clinical relevance of these differences depends on whether the observed values exceed physiological thresholds.

	Mean translation in mm			
Number of cycles	Group A (*n* = 10)	Group B (*n* = 10)	Group C (*n* = 10)	*P*-value
10	1.48 ± 0.33	1.42 ± 0.24	1.65 ± 0.51	0.372
100	1.47 ± 0.31	1.40 ± 0.24	1.52 ± 0.40	0.691
500	1.47 ± 0.29	1.38 ± 0.29	1.51 ± 0.40	0.669
1000	1.49 ± 0.28	1.39 ± 0.31	1.50 ± 0.41	0.718
1500	1.48 ± 0.29	1.40 ± 0.32	1.50 ± 0.41	0.797
2000	1.44 ± 0.28	1.39 ± 0.32	1.49 ± 0.41	0.823

### Changes in the anterior, central, and posterior gaps and step-offs

**Figure 4:**
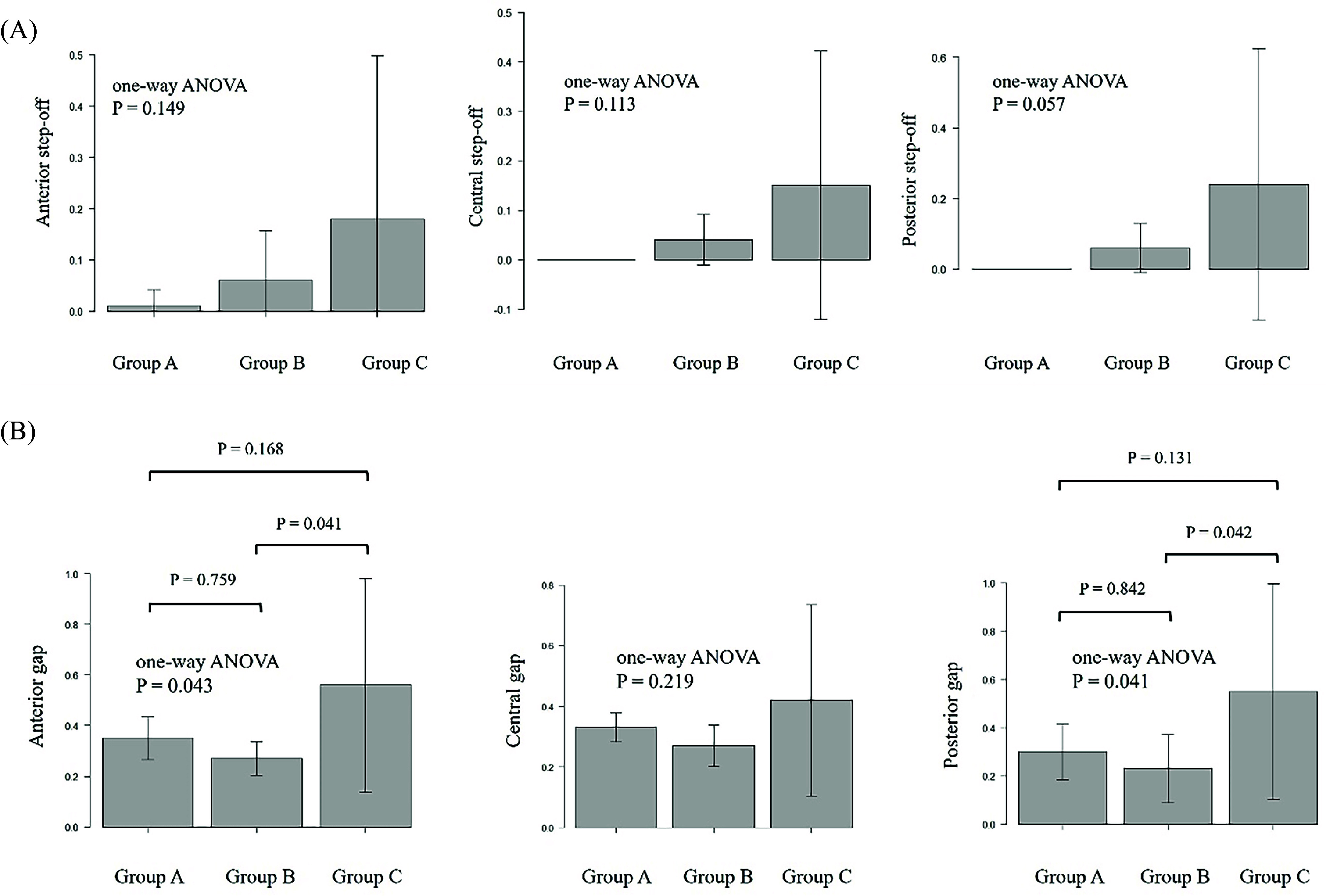
Step-off and gap changes for each of the three groups. A) Step-off changes among the three groups assessed by one-way ANOVA. B) Gap changes among the three groups assessed by one-way ANOVA. These parameters indicate differences in fixation stability and alignment after cyclic loading. One-way ANOVA: one-way analysis of variance.

One-way ANOVA revealed no significant differences in anterior step-off changes among the three groups (group A: 0.01 ± 0.03 mm, group B: 0.06 ± 0.10 mm, group C: 0.18 ± 0.32 mm; *P* = 0.15). Similarly, no significant changes were observed in central step-off changes (group A: 0.02 ± 0.04 mm, group B: 0.04 ± 0.05 mm, group C: 0.15 ± 0.27 mm; *P* = 0.11) and posterior step-off changes (group A: 0.01 ± 0.03 mm, group B: 0.06 ± 0.07 mm, group C: 0.24 ± 0.38 mm; *P* = 0.15) after cyclic loading tests. Post hoc analysis also showed no significant differences between groups A and B, groups A and C, and groups B and C (*P* = 0.82, 0.14, and 0.36, respectively) (Figure 4A).

One-way ANOVA revealed a significant difference in anterior gap changes (group A: 0.35 ± 0.09 mm, group B: 0.27 ± 0.07 mm, group C: 0.56 ± 0.42 mm; *P* = 0.042) and posterior gap changes among the three groups (group A: 0.30 ± 0.12 mm, group B: 0.23 ± 0.14 mm, group C: 0.55 ± 0.45 mm; *P* = 0.04) (Figure 4B). However, no significant differences in central gap changes were observed among the three groups (group A: 0.33 ± 0.05 mm, group B: 0.27 ± 0.07 mm, group C: 0.42 ± 0.32 mm; *P* = 0.22). Post hoc analysis revealed that group B had a lower mean anterior gap than group C (*P* = 0.041), but no significant difference in the anterior gap was observed between groups A and B and groups A and C (*P* = 0.759 and 0.168, respectively). Similarly, group B had a lower mean posterior gap than group C (*P* = 0.042), but no significant difference in the posterior gap was observed between groups A and B and groups A and C (*P* = 0.842 and 0.131, respectively) (Figure 4B).

## Discussion

In this study, we evaluated the biomechanical impact of screw and plate reinsertion into the same holes after implant removal in MTPFs. A significant difference in displacement during cyclic loading was observed only in the 10–100 cycle interval, where post hoc analysis revealed lower displacement in group A than in groups B and C. However, no significant differences were found in other displacement and translation measurements. Clinically, tibial plateau fractures with relatively small gaps or step-offs (up to 4 mm) are considered minimally displaced [12, 15]. Thus, despite statistical significance, the absolute displacement values were small, suggesting minimal clinical impact.

Regarding fracture gaps and step-offs, significant differences were observed in anterior and posterior gap changes, with group B showing lower mean values than group C. However, no significant differences were detected between groups A and B or A and C. These findings indicate that reinserting screws and plates into the same holes may not substantially compromise overall fixation strength.

Previous biomechanical studies have investigated screw reinsertion, particularly in the context of pedicle screws for spine fixation [16–18]. However, specific research on LP fixation after implant removal and reinsertion remains limited [19]. Moreover, previous studies have primarily focused on the pullout strength of reinserted screws [16–19]. In contrast, no studies have examined load application tests using cyclic loading with LP, as identified in our literature search. In the tibia, pullout forces are not typically a primary concern, making load application tests a more clinically relevant research approach.

During screw replacement, over-tightening beyond the maximum insertion torque (MIT) results in screw stripping [20], which can reduce pullout strength by up to 80% [21]. Stripping is particularly common with 3.5-mm cortical screws during osteosynthesis [22], especially in osteoporotic bone [23]. When stripping occurs, a 4.0 cancellous “bailout” screw can be used as a replacement. Ideally, this bailout screw should replicate the original insertion torque of the primary cortical screw. Many surgeons recognize the adequacy of this replacement in clinical practice. However, this has not yet been systematically examined in laboratory settings. Reinserting a 3.5-mm non-locked cortical screw may significantly decrease its MIT [4]. While a bailout cancellous screw can be expected to restore the MIT in normal cortical bone and osteoporotic cancellous bone, it cannot be consistently relied upon to achieve the full insertion torque in all cases [24]. In the present study, we did not find evidence that screw reinsertion leads to fracture instability. Several factors may explain this finding. First, this study employed cyclic loading tests rather than pullout strength tests. Second, the use of LP and locking screws instead of cortical and cancellous screws may have compensated for any strength reduction caused by screw reinsertion. Third, we used porcine models with good bone quality rather than osteoporotic models. Despite these factors, the strength of this study lies in the fact that it is the first to confirm the mechanical strength of reinsertion and refixation with the same plates and screws to the same hole in an MTPF model.

In the treatment of proximal tibia fractures, some studies have reported a significantly higher SSI rate of approximately 10% for these fractures compared with previously reported rates, and patients with SSI had poor outcomes [25, 26]. In the treatment of KOA, the use of medial open wedge high tibial osteotomy (MOWHTO) resulted in earlier rehabilitation, less frequent neurovascular complications, and easier transition to total knee arthroplasty, leading to its increasing popularity, compared with lateral closed wedge high tibial osteotomy [27, 28]. However, MOWHTO is associated with a higher incidence of complications, including patella baja, SSI, delayed union and nonunion, hardware failure, and lateral cortex fractures [29, 30]. Guoxing reported an SSI rate of 4.2% after MOWHTO [31]. If SSI occurs, surgeons may consider implant removal, regardless of whether the bone union has been achieved. During refixation, placing the plate and screw in the same screw hole is thought to weaken the fixation strength. However, no studies have specifically addressed this issue. Our findings might suggest that the reinsertion of the same plate and screws into the same holes does not significantly compromise mechanical stability. Clinically, this implies that in non-osteoporotic bone, surgeons may not need to universally replace screws with larger diameter bailout screws. Instead, careful assessment of bone quality and reinsertion torque is necessary.

However, this study has several limitations. First, a porcine model was used, so the findings may not directly translate to clinical practice. Differences between human and porcine tibiae may influence fixation forces, although porcine knees share many similarities with human knees [6, 7]. Second, there are various types of MTPFs [7]. However, this study focused on only one fracture type, using a previously established fracture model as a reference [12]. We are considering biomechanical studies on other types of medial plateau fractures. Third, this study used LPs without bending. Although bending the LPs could have reinforced the buttresses, we opted not to bend them to maintain consistent stiffness. Fourth, for measuring fracture gaps and step-offs, no intra-examiner errors were detected; however, inter-examiner errors were not assessed. Fifth, only initial structural characteristics were evaluated using specimens with LPs placed medially and anteromedially, so any biological healing response was not considered. Additionally, this study was conducted ex vivo on tibial sections, meaning the results may not fully reflect actual in vivo loading conditions. In a biological knee, loading is not limited to axial forces but also involves rotational movements, resulting in complex three-dimensional motion. Future research should incorporate loading tests that include rotational forces in an ex vivo setting to better replicate physiological conditions or be conducted in vivo. Please let us know if further clarification is needed.

In clinical practice, using the medial placement of LPs and large-fragment LPs for MTPFs may lead to lower postoperative reduction losses than using the anteromedial placement and small-fragment LPs for such fractures.

## Conclusion

Screw reinsertion in the same holes after removal did not significantly compromise the stability of osteosynthesis in this biomechanical model. These findings suggest that reinsertion may be a viable option in revision surgery.

## Data Availability

Data and materials for this study are available from the corresponding author upon reasonable request.
